# 10-year follow-up of interventional electrophysiology: updated German survey during the COVID-19 pandemic

**DOI:** 10.1007/s00392-022-02090-3

**Published:** 2022-09-06

**Authors:** Lars Eckardt, Florian Doldi, Sonia Busch, David Duncker, H. Estner, M. Kuniss, A. Metzner, C. Meyer, H.-R. Neuberger, R. Tilz, F. Voss, D. Steven, P. Sommer

**Affiliations:** 1 Klinik Für Kardiologie II: Rhythmologie, Universitätsklinikum Münster, Albert-Schweitzer Campus 1, 48149 Münster, Germany; 2 II Medizinische Klinik Für Kardiologie, Pneumologie Und Angiologie, Krankenhaus Coburg, Coburg, Germany; 3 Klinik Für Kardiologie Und Angiologie, Zentrum Innere Medizin, Medizinische Hochschule Hannover, Hannover, Germany; 4 Medizinische Klinik Und Poliklinik, Interventionelle Elektrophysiologie, Klinikum Der Universität München, Campus Großhadern, Munich, Germany; 5 Abteilung Kardiologie, Kerckhoff Klinik GmbH, Bad Nauheim, Germany; 6 Klinik Und Poliklinik Für Kardiologie, Universitäres Herz- Und Gefäßzentrum UKE Hamburg, Hamburg, Germany; 7 Klinik Für Kardiologie, Angiologie, Intensivmedizin, cNEP Research Consortium EVK, Düsseldorf, Germany; 8 Klinik Für Kardiologie-Rhythmologie, Klinikum Traunstein, Traunstein, Germany; 9 Medizinische Klinik II (Kardiologie, Angiologie, Intensivmedizin), UKSH, Lübeck, Germany; 10 Innere Medizin III, Krankenhaus der Barmherzigen Brüder Trier, Trier, Germany; 11 Klinik III Für Innere Medizin, Abteilung Für Elektrophysiologie, Herzzentrum Uniklinik Köln, Cologne, Germany; 12 Klinik Für Elektrophysiologie/Rhythmologie, Herz Und Diabeteszentrum NRW, Bad Oeynhausen, Germany

**Keywords:** Interventional electrophysiology, Catheter ablation, Survey, Training requirements

## Abstract

**Introduction:**

This study provides an update of survey-based data providing an overview of interventional electrophysiology over the last decade. Overall infrastructure, procedures, and training opportunities in Germany were assessed.

**Methods:**

By analyzing mandatory quality reports, German cardiology centres performing electrophysiological studies were identified to repeat a questionnaire from 2010 and 2015.

**Results:**

A complete questionnaire was returned by 192 centers performing about 75% of all ablations in Germany in 2020. In the presence of the COVID-19 pandemic, a total of 76.304 procedures including 68.407 ablations were reported representing a 38% increase compared to 2015. The median number of ablations increased from 180 in 2010 to 377 in 2020. AF was the most common arrhythmia ablated (51 vs. 35% in 2010). PVI with radiofrequency point-by-point ablation (64%) and cryo-balloon ablation (34%) were the preferred strategies. Less than 50 (75) PVI were performed by 31% (36%) of all centres. Only 25 and 24% of participating centres fulfilled EHRA and national requirements for training centre accreditation, respectively. There was a high number of EP centres with no fellows (38%). The proportion of female fellows in EP increased from 26% in 2010 to 33% in 2020.

**Conclusion:**

Comparing 2020, 2010 and 2015, an increasing number of EP centres and procedures were registered. In 2020, more than every second ablation was for therapy of AF. In the presence of an increasing number of procedures, training opportunities were still limited, and most centres did not fulfill recommended EHRA or national requirements for accreditation.

**Graphical abstract:**

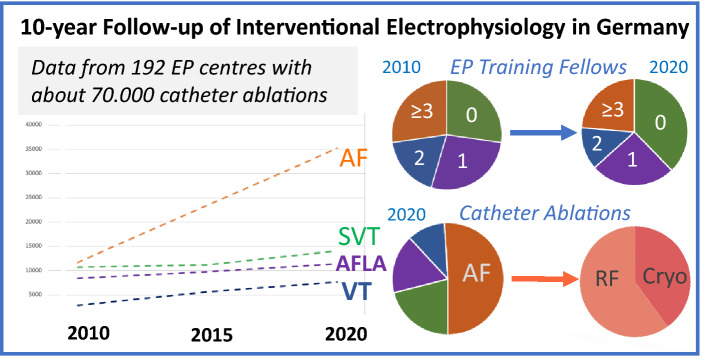

## Introduction

Over the last decades, cardiac electrophysiology has become a pivotal subspecialty of cardiology with growing numbers of catheter ablations every year [1]. In many patients with supraventricular tachycardias (SVT) or atrial fibrillation (AF), catheter ablation is considered first-line therapy [2, 3]. The gradual increase in the number of yearly performed catheter ablations is, e.g. portrayed in mandatory quality reports based on the German operational and procedural key system (OPS) with currently about 90,000 catheter ablations in Germany each year [4, 5].

To ensure overall quality, safety, and optimal patient care national and international standards as well as trained specialists in the field of cardiac electrophysiology are encouraged to match this development. In order that aspiring physicians in the field of cardiac electrophysiology receive proper training as heart rhythm specialists, national and international cardiology societies have developed training programs and curricula [6–9].

To provide an overview and assess the current national status of physician training and patient care in cardiac electrophysiology including infrastructure, training conditions, and ablation procedures, we initiated this survey in 2010 [10] and performed a 5-year follow-up in 2015 [11]. This multi-centre observational study provides a second longer follow-up and overview of a decade of electrophysiological patient care and training comparing data to previous surveys from 2010 and 2015. It is of particular interest as it presents data of a time period in which the worldwide COVID-19 pandemic enforced lock-down measures with cancellation of many elective catheter ablations.

## Methods

Consulting the national legally mandatory quality reports of German hospitals, 340 centres were identified currently performing electrophysiological studies with the following reported OPS (operation and procedure code): 8–835.2 (radiofrequency (RF) ablation), 8–835.3 (irrigated RF ablation), 8–835.4 (ablation with other energy sources), 8–835.9 (MESH ablation), 8–835.a (cryo-ablation), and 8–835.8 (ablation with 3-D mapping). (https://www.dimdi.de/dynamic/de/klassifikationen/ops/anwendung/zweck/index.html).

As more than one OPS code can be reported for a single ablation procedure (e.g., radiofrequency ablation plus 3D mapping-based ablation), the number of OPS given is not equal to the number of procedures performed. Centres coding for less than 30 ablation procedures a year were excluded to prevent the accidental inclusion of centres employing external electrophysiologists or coding OPS for externally performed procedures.

Upon identification of the centres, we contacted the cardiology or interventional electrophysiology department by e-mail and/or phone to complete the same questionnaire that was utilized in previous surveys from 2010 [10] and 2015 [11].

Among the included parameters in the questionnaire were: type of hospital; staff numbers and functions in cardiology and electrophysiology, gender aspects, infrastructure, number and types of EP procedures, techniques used, imaging modalities, presence of or distance to cardiac surgery. Furthermore, more detailed information on protection methods of the esophagus during AF ablation was requested. Gathered data were anonymized and consequently analyzed using R-Studio Version 1.4.1106 (R. RStudio, PBC, Boston, MA).

## Results

**Fig. 1:**
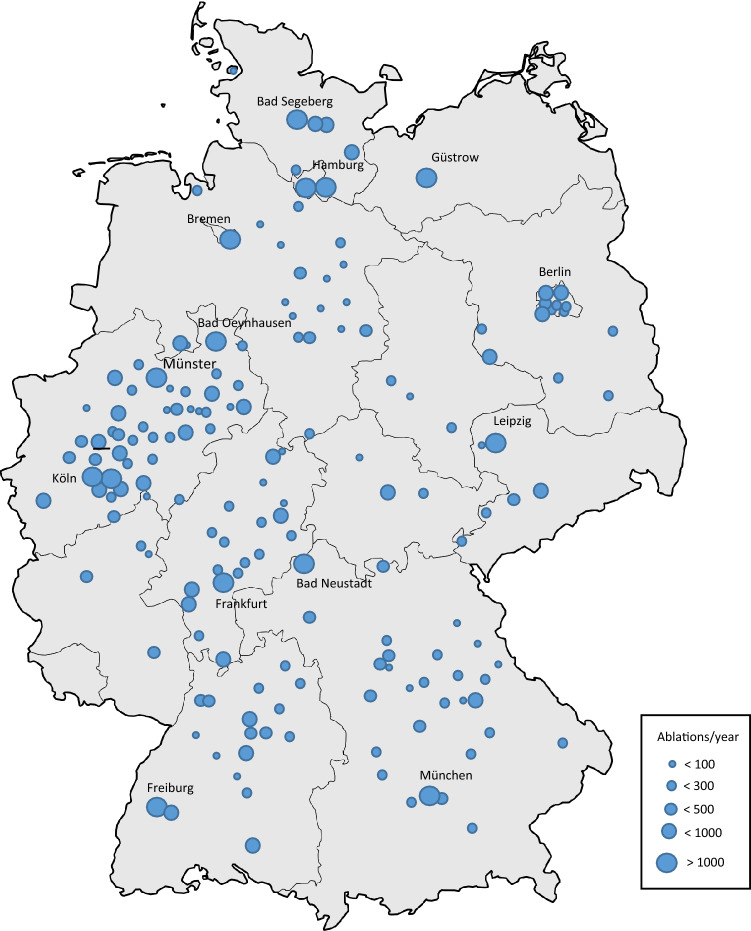
Map of Germany depicting included electrophysiologic centres according to the number of ablations performed each year

Of all the centres, coding more than 30 ablation procedures per year, 192 (56%) answered the survey and were included in this analysis (Fig. 1). Responding centres included 34 (18%) university hospitals, 137 (71%) teaching hospitals (non-university hospitals involved in training of medical students), 19 (10%) non-teaching, and 2 (1%) private medical practices performing ablations in adjoining hospitals.

### The structure of interventional electrophysiology

The electrophysiological departments were mainly part of a cardiology clinic (90%) with only 19 EP centres (11%) being independent with their own budget. A total of 106 centres (55%) were certified training centres for cardiac electrophysiological procedures by the German cardiac society (DGK). Heads of cardiological departments of 31 centres (16%) counted invasive electrophysiology as their main area of expertise. In 148 centres (77%), at least one catheter laboratory was exclusively used for invasive electrophysiology over 90% of the time. Thirty-five centres (18%) used two laboratories predominantly for EP procedures. 3-D mapping systems (CARTO^®^ *n* = 104; NavX^®^ *n* = 106; Rhythmia^®^ *n* = 29; CARTO^®^ and NavX^®^ *n* = 47) were available in 110 (57%) centres.

101 centres (53%) used the catheter laboratory also for all electrical device implantations, 12 (6%) centres in more than 50% of cases and 45 (23%) centres in less than 50% of cases. In the remaining centres (*n* = 34; 18%), device implantations were exclusively performed in operating rooms. The primary operator implanting these devices was a cardiologist in 147 (77%) centres and a surgeon in 8 (4%). Both cardiologists and surgeons performed these procedures in the remaining 36 (19%) EP centres.

### Physicians involved in electrophysiology

Altogether there were 219 heads (female: *n* = 9; 4%) of departments with 27 centres (14%) having more than one head of department (including head for interventional cardiology and electrophysiology) (Table 1). Furthermore, 1424 consultants (“Oberarzt”, female: *n* = 338; 24%) and 3441 physicians in training (female: *n* = 1652; 48%) were employed. A total of 403 EP consultants (female: “Oberärztin” *n* = 75; 19%) were employed with 36 (19%) centres having only one and 146 centres (76%) having two or more EP consultants in their team. EP Consultants from 139 centres (72%) also performed coronary interventions (Table 1).

**Table 1:** Comparison of 2010, 2015 and 2020 survey data on structure and training in electrophysiology in Germany

	2010 (%)	2015 (%)	2020 (%)
Responding centres	122	131	192
EP part of a cardiology department	111 (91)	117 (89)	173 (90)
Independent EP (own budget)	11 (10)	14 (12)	19 (11)
More than one head of department	12 (10)	35 (27)	29 (15)
Heads of department [female]	149 [3 (2)]	166 [4 (2)]	219 [9 (4)]
Consultants “Oberärztin/arzt” [female]	764 [109 (14)]	988 [201 (21)]	1424 [338 (24)]
Centres with only 1 electrophysiologist	30 (25)	8 (6)	7 (4)
Fellows in cardiolgy/EP [female]	2365 [1044 (44)]	2801 [1371 (49)]	3441 [1652 (48)]
Fellows in EP only [female] (%)	235 [61 (26)]	291 [112 (38)]	432 [144 (33)]
EP consultants [female] (%)	193 [19 (10)]	276 [48 (17)]	403 [75 (19)]
One EP consultant (%)	49 (40)	28 (22)	36 (19)
Two or more EP consultants (%)	55 (45)	88 (67)	146 (76)
Centres with EP consultants also performing PCI (%)	94 (77)	83 (63)	139 (72)
Centres with no EP fellows* (%)	42 (34)	41 (33)	73 (38)
Centres with 1 EP fellow (%)	29 (24)	28 (22)	46 (24)
Centres with 2 EP fellows (%)	19 (16)	20 (16)	22 (11)
Centres with 3 or more EP fellows (%)	32 (26)	37 (29)	51 (27)
Primary operators for ablation [female] (%)	309 [28 (9)]	403 [73 (18)]	549 [126 (23)]
Less than 40 years old (%)	122 (39)	163 (40)	203 (37)
Between 40 and 50 years (%)	152 (48)	166 (41)	214 (39)
Older than 50 years (%)	35 (2)	74 (18)	132 (24)
Worked part-time (%)	7 (2)	32 (8)	53 (10)
Centres with at least 2 physicians during ablation procedures (%)	71 (58)	86 (66)	115 (60)

Values are *n* or *n* (%)

*EP* electrophysiology, *PCI* percutaneous coronary intervention

*According to a position paper by the DGK (8), 75 AF ablations per year are required, which is fulfilled by 122 (64%) and results in only 36 (19%) centres fulfilling all DGK requirements

**Fig. 2:**
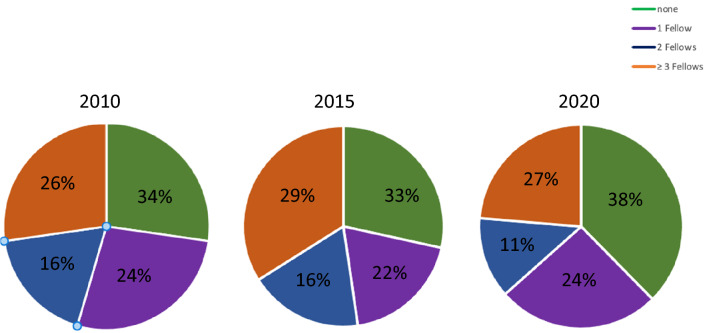
Number of Training Positions in 2010, 2015 and 2020 per Center Performing Interventional Electrophysiology

For EP fellows, there were a total of 432 (female: *n* = 144: 33%) training positions reported. In 46 (24%) centres, only one fellow was trained as a heart rhythm specialist. No less than 2 fellows were employed in 22 (11%) centres and at least 3 or more fellows in 51 (27%) centres. In contrast, 72 (38%) centres had no EP fellows (Fig. 2). As primary operator, 549 (female: *n* = 126; 23%) EP consultants performed catheter ablations with only one EP consultant present in the cardiological team in 7 centres (4%). Of these primary operators, 203 (37%) were less than 40 years old, 214 (39%) between 40 and 50, and 132 (24%) more than 50 years old; 53 (10%) worked part-time.

A median number of 377 catheter ablations per centre were performed in 2020 with two or more physicians present throughout most ablation procedures in 134 (70%) centres (Table 2). Less than 100 catheter ablations were performed at 33 (17%) centres, and in 108 (56%) centres, at least 200 ablations were performed. At least 50 (75) PVI were documented in 133 (69%) centres (*n* = 122; 64%, respectively); 59 centres (31%) performed less than 50 PVI and 25 (13%) centres were not ablating AF at all.

**Table 2:** Comparison of 2010, 2015 and 2020 survey data on number and technical aspects of catheter ablation procedures

	2010 (%)	2015 (%)	2020 (%)
Responding centres	122	131	192
Median number of ablations	180	297	377
Centres with less than 100 ablations (%)	32 (26)	19 (15)	33 (17)
Centres with at least 200 ablations (%)	59 (48)	91 (69)	108 (56)
Centres with at least 50 PVI (%)	65 (53)	105 (80)	133 (69)
EP procedures	40,735	59,033	76,304
Catheter ablations	33,420	49,356	68,407
Paroxysmal SVT ablations (%)	10,726 (32)	11,221 (22)	14,045 (21)
Arial flutter ablations (%)	8396 (25)	9749 (20)	11,428 (17)
Ventricular tachycardia/VPC (%)	2837 (8)	5621 (11)	7641 (11)
Atrial fibrillation ablations (%)	11,685 (35)	23,441 (47)	35,193 (51)
Centres with trans-septal approach for left-sided accessory pathways (%)	55 (56)	83 (63)	131 (68)
Centres performing ablation of left ventricular VT (%)	81 (66)	111 (85)	149 (78)
Centres performing no VT ablations (%)	27 (22)	18 (14)	45 (23)
Primary retrograde approach for left ventricular VT ablations (% of VT centres)	55 (68)	51 (46)	61 (41)
Primary trans-septal approach for left ventricular VT ablations (% of VT centres)	26 (32)	60 (54)	88 (59)
Centres performing epicardial VT ablations (%)	15 (12)	38 (29)	44 (23)
Patient consent for ablation before hospital admission with ablation on day of admission (%)			
Always	22(18)	22 (17)	39 (20)
> 50%	42 (34)	44 (34)	78 (41)
< 50%	17(14)	31(24)	50 (26)
< 10%	41(34)	34 (26)	25 (13)

Values are *n* or *n* (%)

*PVI* pulmonary vein isolation, *SVT* supraventricular tachycardia, *VPC* ventricular premature complex, *VT* ventricular tachycardia

### Procedural data

The reporting 192 centres performed a total of 76.304 EP procedures including 68.407 catheter ablations in 2020. Most of the centres obtained patient consent already before hospital admission: 39 (20%) centres in all cases; 78 (41%) in over 50% of the cases. (Table 2).

**Fig. 3:**
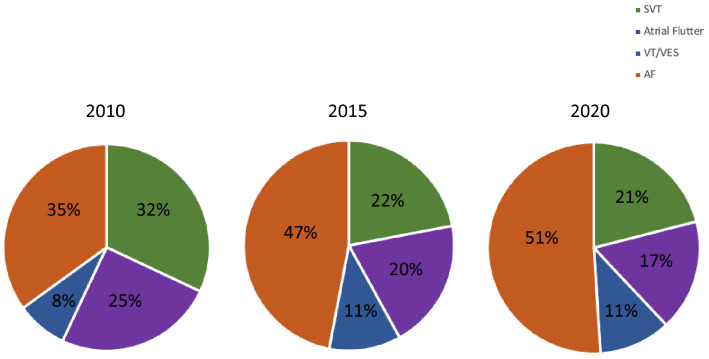
Catheter ablations in Germany, 2010 (*n* = 33,420); 2015 (*n* = 49,356) and 2020 (*n* = 68,407)

The most frequent arrhythmia treated by catheter ablation was AF (*n* = 35.193; 51%) followed by SVT (*n* = 14.045; 21%), atrial flutter (*n* = 11.428; 17%), and ventricular tachycardias (*n* = 7.641; 11%) (Fig. 3). Left-sided accessory pathways were ablated by 176 (92%) centres, out of these, 140 centres (80%) primarily used a transseptal and 36 of the centres (20%) a retrograde approach. 149 centres (78%) performed left-sided VT ablations with the majority of these centres (*n* = 88; 59%) using a trans-septal and 61 centres (41%) a retrograde approach to reach the left ventricle. Of note, VT were not ablated in 43 (22%) centres. If necessary, 44 centres (23%) reported to perform epicardial ablations (Table 2).

**Fig. 4:**
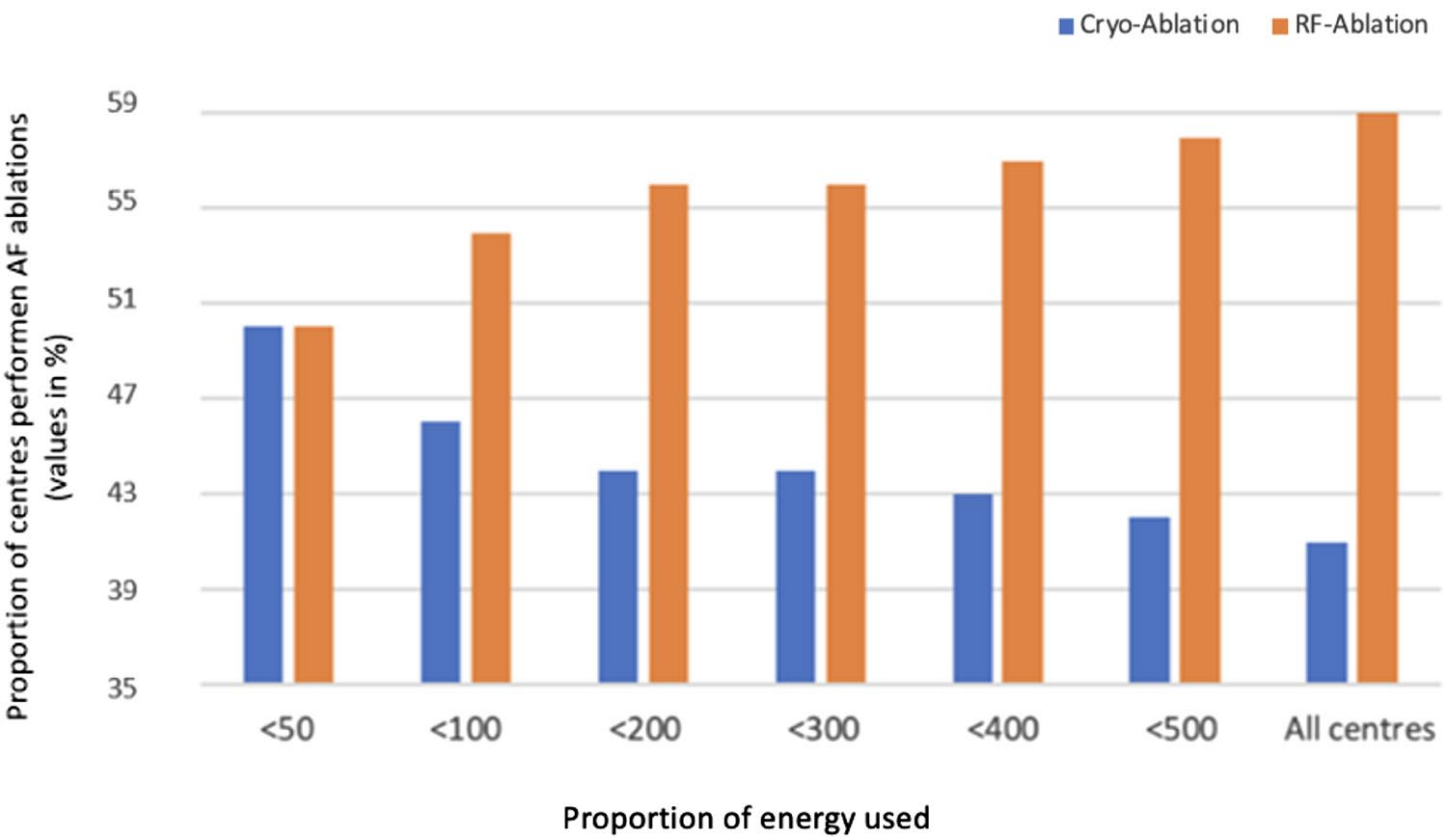
Proportion of centres performing AF ablations with radiofrequency (RF) point-by-point ablation versus Cryo-balloon ablation in relation to the number of AF ablations performed per centre in 2020

The energy source predominantly used by the 167 centres (87% of all participating centres) ablating AF was point-by-point radiofrequency current with 64% of all PVI vs. 34% cryo-balloon ablations (Table 3). The proportion of cryo-balloon ablation clearly correlated with the centres´ total number of PVI, the larger the volume, the higher the proportion of RF ablation (Fig. 4). In persistent AF, the primary ablation strategy reported was PVI in 147 centres (88%) with a minority of the centres performing PVI plus linear ablation (*n* = 4; 2%) or substrate modification using, e.g. defragmentation (*n* = 17; 10%). In 72 centres (43%), imaging before AF ablation was routinely performed (MRI in 11 (7%), CT in 35 (21%), rotational angiography in 17 (10%); 3-D Echo in 9 (12%)). Consecutive atrial arrhythmias after AF ablation were ablated in 147 (77%) of participating centres. Sedation with propofol was the preferred standard approach (95%). Only a small number of centres (*n* = 7; 4%) performed ablations under general anesthesia.

**Table 3:** Comparison of 2010, 2015 and 2020 survey data on technical aspects of catheter ablation of atrial fibrillation

	2010 (%)	2015 (%)	2020 (%)
Responding centres	122	131	192
Strategy for AF ablation			
Centres performing AF ablations	**99 (81)**	**123 (94)**	**167 (87)**
Atrial fibrillation ablations	11,685 (35)	23,441 (47)	35,193 (51)
Radiofrequency point-by-point ablations	NA	14,728 (63)	22,558 (64)
Cryo-ablations	NA	7781 (33)	12,042 (34)
Other energy sources/techniques	NA	932 (4)	586 (2)
Centres performing ablations of consecutive left atrial arrhythmias after PVI (% of all centers)	74 (61)	106 (81)	147 (77)
Preferred ablation strategy for persistent AF (% of centres performing AF Ablations)			
(only) PVI	NA	102 (83)	147 (88)
PVI plus linear ablation	NA	11 (9)	4 (2)
PVI plus defragmentation and/or substrate modification	NA	10 (8)	17 (10)
Surgical back-up and AF surgery (% of centres performing AF ablations)			
In-house surgical back-up	44 (44)	55 (45)	64 (38)
Centres performing surgical AF ablations	41 (41)	37 (30)	44 (26)
Centres performing stand-alone surgical AF abl	10 (10)	11 (9)	10 (6)
Imaging before AF ablation (% of centres performing AF Ablations)			
Centres routinely performing LA imaging before AF ablation	59 (60)	61 (50)	72 (43)
MRI	14 (14)	16 (13)	11 (7)
TCT	43 (43)	38 (30)	35 (21)
Rotational angiography	2 (2)	7 (6)	17 (10)
Sedation/anaesthesia for AF ablations (% of centres performing AF Ablations)			
Centres using general anesthesia during AF ablations	6 (6)	3 (2)	7 (4)
Sedation with propofol	54 (55)	92 (75)	159 (95)
Sedation without propofol	35 (35)	28 (23)	8 (5)
Protection of the esophagus during AF ablations			
AF ablations centers using strategies for special protection of the esophagus	NA	96 (78)	147 (88)
Energy reduction at the posterior wall	NA	66 (54)	114 (68)
Use of esophageal temperature probes	NA	52 (42)	91 (54)
Use of H_2_ blockers post ablation	NA	85 (69)	130 (78)

Values are *n* or *n* (%)

*AF* atrial fibrillation, *CT* computed tomography, *LA* left atrial, *MRI* magnetic resonance imaging, *NA* not applicable, *PVI* pulmonary vein isolation

Though rare, an atrio-esophageal fistula remains one of the most feared late complications after PVI because of its often lethal outcome [12]. Therefore, the vast majority of centres (88%) reported using strategies for esophageal protection including: prescribing H_2_ blockers (78%) after ablation [13], reducing energy while ablating along the posterior wall (68%) and the use of esophageal temperature probes (54%) [14–16].

Cardio-surgical back-up was available in-house in 64 (38%) of the centres performing AF ablations. If not available in-house, the distance to the next hospital having a cardio-surgical unit ranged from 1 to 150 km (mean: 35 ± 31 km). Surgical AF ablations were performed in 44 (26%) centres with 10 (6%) centres performing surgical AF ablations as stand-alone operations.

### Training centre requirements

The requirements to be accredited as an EP training centre according to the European Heart Rhythm Association (EHRA) and the German Cardiac Society (DGK) are illustrated in Table 4. Only a quarter (*n* = 48) of the responding centres fulfilled the requirements provided by the EHRA or DGK (*n* = 47; 24%; for the requirement of 75 AF ablations/per year *n* = 36 (19%)).

**Table 4:** Curriculum heart rhythm specialists: Training centre requirements and reality (Germany 2020)

Parameter	EHRA requirements	Centers fulfilling EHRA requirements	DGK requirements	Centres fulfilling DGK requirements
Physicians present during ablation	–		2	31 (16%) (always)^a^ 134 (70%) (most cases)
No. EP procedures/year	200	112 (58)	250	106 (55)
No. ablations/year	150	119 (62)	200	108 (56)
No AF ablations/year	–	–	50 [75*]	133 (69) [122 (64)]
3D mapping system	Yes	110 (57)	Yes	110 (57)
Cardio-surgical unit	Yes	64 (38)	No	–
All requirements		48 (25)		47 (24)

Values are *n* or (*n*%). Requirements are defined according to guidelines and curricula as published (6–9)

*3D* 3-dimensional, *DGK* German Society of Cardiology, *EHRA* European Heart Rhythm Association

^a^Under the requirement that always 2 physicians are present during an ablation only 31 centres (16%) would have fulfilled DGK requirements

*According to a position paper by the DGK (8), 75 AF ablations per year are required, which is fulfilled by 122 (64%) and results in only 36 (19%) centres fulfilling all DGK requirements

## Discussion

Reporting data from German centres performing electrophysiological studies, this multi-centre observational study is able to describe clear trends in electrophysiology over the recent decade comparing data from 2010 [10], 2015 [11], and 2020. Most contacted clinics responded with a complete questionnaire. Collectively, there were 68.407 catheter ablations reported by the responding centres in 2020 illustrating a 39 and 105% increase in yearly performed ablations compared to survey data from 2015 [11] and 2010 [10], respectively. This is in line with an increase in the number of hospitals performing EP studies in Germany and was observed despite the presence of the COVID-19 pandemic with many weeks of lock-down and cancellation of elective EP procedures in most centres.

As training requirements differ not only in Europe but also in the U.S. it is difficult to determine an exact number of necessary ablation procedures needed to be an experienced EP centre [17]. Reference publications are the curricula published by the German cardiac society (DGK) [7, 8] and the European Heart Rhythm Association (EHRA) [6] as well as the 2017 HRS/EHRA/ECAS/APHRS/SOLAECE expert consensus statement on catheter and surgical ablation of atrial fibrillation [17]. These recommendations are very similar, except the required ablation numbers in Europe being slightly higher. The EHRA (DGK) recommend that an EP centre ought to have a (moderate) quantity of at least 200 (250) EP studies and at least 150 (200) catheter ablations a year which was, however, fulfilled by only 58% (55%) of the responding centres. Besides, the EHRA requires a centre to have a cardio-surgical unit which was present in only 38% of the participating German centres. Altogether, only a quarter of responding centres fulfilled all EHRA or DGK criteria. Of note, only 16% of the centres fulfilled the requirement of the DGK of always having two physicians present during catheter ablation procedures. Analyzing these results and comparing them with data from 2010 and 2015, there is still a relevant need to enhance the quality of EP physician training and for collaboration between centres to provide high-quality electrophysiological patient care. Because many centres do not fulfill requirements set by the EHRA and/or DGK, one can assume there is a scarcity of training opportunities for physicians aspiring a career in EP. However, a centre accreditation by neither institution reflects the capacity of a single operator and is only supposed to show which centre would have met certain requirements agreed upon by a committee of experienced electrophysiologists.

Very recently, a survey of members of the “Young DGK” (median age 33 ± 3.3 years) regarding training opportunities for cardiology was published [18]. The majority wished more electrophysiological training opportunities with 50% of cardiological fellows reporting not to receive any EP training [18]. These results directly reflect to our survey with still more than a third (38%) of the responding centers reporting to have no EP fellows at all. This has remained almost unchanged throughout the last decade (2010: 34%; 2015: 33%). Thus, the present situation of German cardiac electrophysiology clearly illustrates (1) an increasing number of catheter ablations in the presence of (2) the necessity of more and better training opportunities.

In the presence of increasing ablation numbers with growing complexity and novel ablation technologies, a high degree of sub-specialization is needed to perform these ablations. It is therefore surprising that (1) only 11% of the centres have an independent EP department (with/without its own budget) and (2) the majority of EP consultants also performs PCI on a routine basis. This proportion even increased in comparison with data from 2015 (63 vs. 72%). One may speculate that these aspects as well as the above-mentioned limited training opportunities require more dedicated independent EP centres in the future.

Despite an overall increase of female physicians in most cardiological specialties, only less than 10% choose a career in EP [19]. Addressing this disparity, a survey by Abdulsalam et al. determined factors influencing physicians in training and career planning. Of the responding participants having an interest in EP, the vast majority that ultimately chose to train as a heart rhythm specialist were men (84 vs. 16%). As potential reasons women reported, e.g. radiation concerns and a perceived “old boys’ club” culture with discrimination/harassment concerns [20]. This issue is also addressed by a survey of Estner et al. [21] showing a large gap between male and female physicians in training (63 vs. 37%) as well as consultants (86 vs. 14%). This corresponds to results from our national survey showing that the proportion of female fellows as well as female EP consultants remain distinctly low with even a decrease in female EP fellows as compared to 2015 (38%; 2020: 33%) and an almost unchanged number of employed female EP consultants (2015: 17%; 2020: 19%). Addressing this issue and improving the training and work environment (e.g., working part-time for both genders, childcare support) will be pivotal to change this disparity in the future. Besides, implementing certain mentorship programs would be of great interest.

**Fig. 5:**
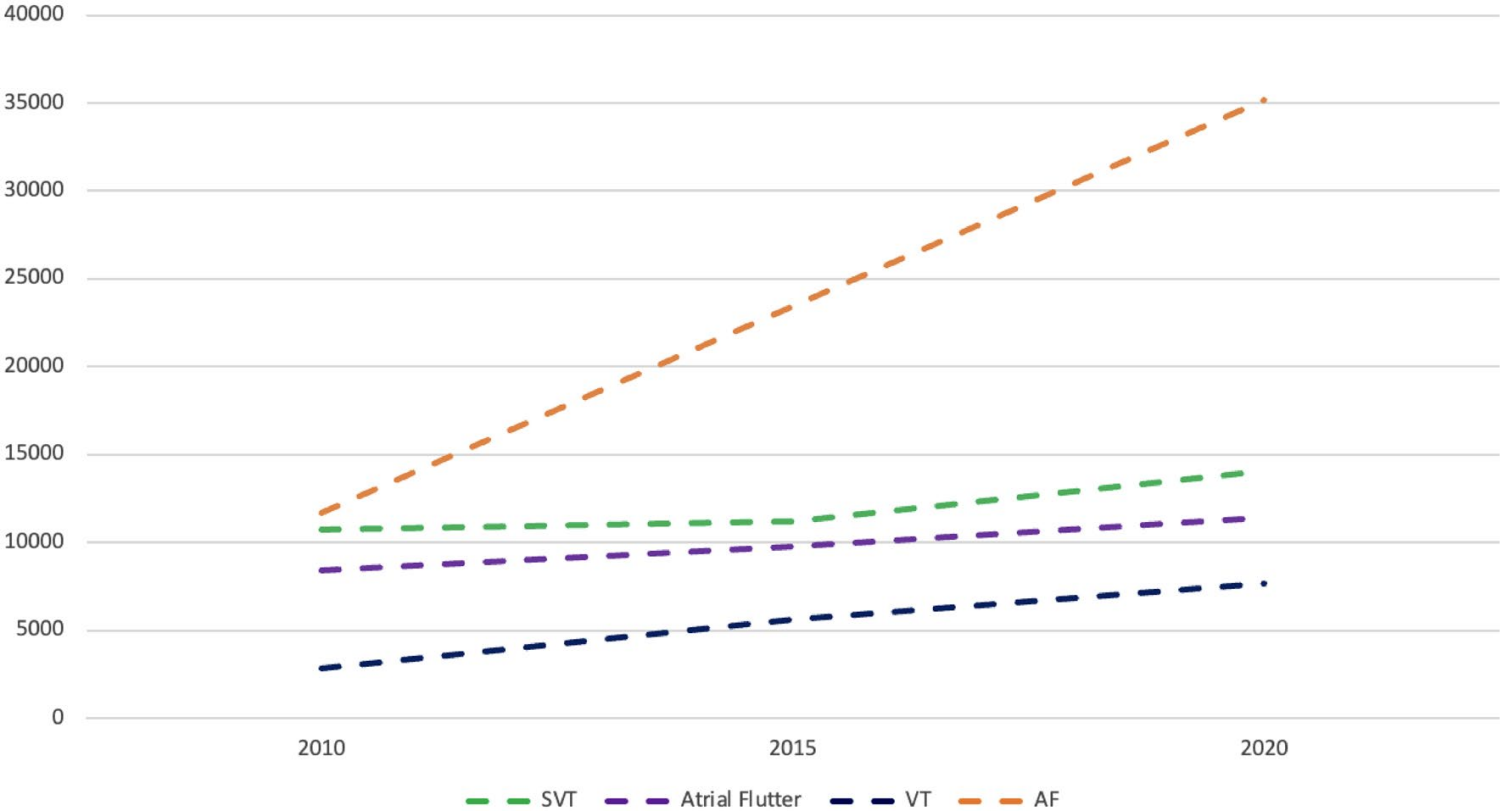
Comparison of the number of SVT, atrial flutter, VT, and AF ablation procedures from 2010, 2015 and 2020 in Germany

As it was seen in 2010 and 2015, PVI remains the most performed catheter ablation procedure even showing an increase in number compared to prior results (2010: 35%; 2015: 47%, 2020: 51%). Considering that during the COVID-19 pandemic more elective PVI were cancelled than urgent ablations such as VT ablations, the true number of scheduled PVI may have been even higher. Nevertheless, the trend of an un-proportional increase in PVI as compared to all other ablation procedures over the last decade is demonstrated by survey comparisons from 2010, over 2015 to 2020 (Fig. 5). In contrast to AF, the number of supraventricular tachycardia (SVT) and atrial flutter ablations remained relatively constant over the years with 22% (32%) and 20% (25%) in 2015 (2010) and 21 and 17% in 2020, respectively. Following the trend in AF ablations and the demography of western countries, one would not be surprised if the next decade will result in PVI accounting for 2/3 of all catheter ablations. Of note, no relevant change is seen regarding the proportion of RF versus cryo-ablations. Most ablations were performed with point-by-point RF ablation (2015: 63%; 2020: 64%) as compared to the cryo-balloon technology (2015: 33%; 2020: 34%). Besides, we could clearly show the less experienced a centre is the more the cryo-balloon is used (Fig. 3). This is in line with the observations of a relevant and increasing portion of centres not ablating consecutive left atrial arrhythmias after PVI compared to 2015 (19 vs. 23% in 2020) [11]. This also most probably reflects the lack of experienced electrophysiologists able to treat consecutive left-sided atrial arrhythmias and the increased use of the technically less demanding cryo-balloon-based ablation by less experienced centres [22]. The STAR AF II Trial [23] and a recent sub-study by Sanchez-Somonte et al. [24] showed that even patients with complete linear block and/or ablation of fractionated electrograms after PVI did not have a better outcome regarding recurring AF. This correlates to our analysis seeing most centres performing PVI only as their first treatment approach for patients with persistent AF as recommended by current guidelines.

As the number of EP procedures increases each year, our observational study is supposed to offer interesting insights into current electrophysiological training and treatment concepts and may help recognizing certain issues that need to be addressed in the future. Besides, further studies setting safety, efficacy, and overall treatment quality in relation to the amount of EP procedures performed per year per centre would give interesting insights and may offer perspectives regarding patient care and physician training.

## Limitations

Certainly, as in the previous studies from 2010 and 2015, not all centres performing EP studies responded and as coding data are not continuously reliable probably not all centres were identified. Nevertheless, our study does include most centres as well as ablations (75%) performed in Germany in 2020 and gives the chance to observe trends over a decade of electrophysiological advances. As the responding centres account for most ablations performed in 2020, smaller clinics might not be well represented in this survey, leading to the possibility of a slight over-estimation of median number of ablations per centre. To prevent the over-estimation of small centres where fewer catheter ablations are performed, we excluded centres coding for less than 30 ablations per year. This again might over-estimate the percentage of possible training centres fulfilling all requirements by the DGK and EHRA. Data about complications and specific outcome would have been of interest (e.g., safety of certain procedures corresponding to the amount of performed procedures a year). But as this survey was devised to assess structural conditions in electrophysiological patient care and physician training, these data are not available.

## Summary

The present multi-centre observational study demonstrates a distinct rise in the need for electrophysiological treatment with increasing numbers of EP centres and performed ablation procedures as compared to 2010 and 2015. Only about a quarter of the centres fulfilled requirements of the EHRA and DGK for EP training centres, respectively. Training positions for physicians in electrophysiology have not adapted to this rising demand and have remained constant over the years. Women are still only scarcely represented in the field of interventional electrophysiology. PVI with point-by-point radiofrequency current (RF) as the mainly used ablation strategy remains the most performed ablation.

## Abbreviations

ACC: American College of Cardiology
AF: Atrial fibrillation
AHA: American Heart Association
CT: Computer tomography
DGK: German Cardiac Society
DRG: Diagnosis-related groups
EHRA: European Heart Rhythm Association
EP: Electrophysiology
ESC: European Society of Cardiology
HRS: Heart Rhythm Society
MRI: Magnetic resonance imaging
OPS: Operation and procedure code
PVI: Pulmonary vein isolation
RF: Radiofrequency
SVT: Supraventricular tachycardia
VT: Ventricular tachycardia
PCI: Percutaneous coronary intervention
CFE: Complex fractionated electrograms
